# Unveiling the intricacies: small interfering RNA targeting Snail-1 unravels dynamics in endometrial carcinoma cell behavior

**DOI:** 10.3389/fonc.2025.1567493

**Published:** 2025-06-12

**Authors:** Feng Li, Yuanyuan Zhi, Yinghui Wang, Shaik Althaf Hussain, Turki Mayudh Alrubie, Ping Yang

**Affiliations:** ^1^ Department of Obstetrics and Gynecology, Xi’an Third Hospital, Xi’an, China; ^2^ Department of Gynecology, Shandong Provincial Maternal and Child Health Care Hospital, Jinan, China; ^3^ Department of Gynecology, The People’s Hospital of Laoling, Dezhou, China; ^4^ Department of Zoology, College of Science, King Saud University, Riyadh, Saudi Arabia; ^5^ Department of Obstetrics and Gynecology, The Third Affiliated Hospital of Xi’an Medical University, Xi’an, China

**Keywords:** endometrial carcinoma, Snail-1, siRNA, metastasis, treating

## Abstract

**Background:**

Investigated within the endometrial carcinoma (EC) context, Snail-1 emerges as a pivotal transcription factor governing invasion and metastasis by orchestrating epithelial-to-mesenchymal transition (EMT). Employing small interfering RNA (siRNA) to silence Snail-1 expression in the HEC-1A cell line, this study explored the repercussions on the expression of genes implicated in metastasis, cellular cytotoxicity, apoptosis, and migration.

**Methods:**

HEC-1A cells were transfected with Snail-1-specific siRNA. Quantitative Real-time PCR was utilized to quantify the mRNA levels of *Snail-1*, Matrix metalloproteinase-9 *(MMP-9), Vimentin, E-cadherin*, *Notch1, ERK, AKT*, and *miR-34a*. Western blot analysis was also performed to ascertain alterations in Snail-1, MMP-9, Vimentin, E-cadherin, and Notch1 protein levels. Cytotoxicity of transfected cells was assessed via the MTT assay, while flow cytometry was employed to measure apoptosis. Migration was evaluated using a wound healing assay.

**Results:**

Transfection with 60 pmol/mL of Snail-1-specific siRNA significantly reduced Snail-1 expression at both the mRNA and protein levels. This was accompanied by decreased MMP-9, Vimentin, and Notch1 expression and increased E-cadherin expression, all confirmed at both transcript and protein levels. Furthermore, gene expression analysis revealed a downregulation of *ERK* and *AKT* mRNA levels and an upregulation of miR-34a. Moreover, transfection correlated with increased apoptosis and decreased migration of treated HEC-1A cells.

**Conclusion:**

The study emphasizes the significant influence of Snail-1 on EMT in EC cells, thereby impacting apoptosis and metastasis. Targeted silencing of Snail-1 through specific siRNA emerges as a promising therapeutic approach in treating EC.

## 1 Introduction

Endometrial carcinoma (EC) stands as a significant malignancy within the female reproductive system, exhibiting a concerning rise in both incidence and mortality rates (1). Diagnosis often occurs at localized stages (I or II according to FIGO criteria), boasting 5-year survival rates ranging from 74% to 91% (2). However, survival rates plummet to 57–66% and 20–26% for patients diagnosed at stages III and IV, respectively (3). While various treatments like hormonal therapy, surgery, chemotherapy, and radiation showed efficacy in localized cases, options remain limited once metastasis occurs (4).

Understanding the molecular mechanisms driving progression from localized to metastatic EC is crucial. Epithelial-mesenchymal transition (EMT) is a pivotal process orchestrating migration, metastasis, and invasion in malignant cells (5, 6). EMT endows epithelial cells with mesenchymal traits, facilitating detachment from the primary tumor site and enabling invasive dissemination (7). E-cadherin loss is a hallmark of EMT-associated changes, with EMT regulators like Snail-1 playing a pivotal role (8).

Snail-1, encoded by the *SNAI1* gene on chromosome 20, is a zinc-finger transcription factor known for its role in transcriptional repression. By suppressing E-cadherin, Snail-1 drives EMT, influencing mesenchymal characteristics such as migration and metastasis (9, 10). Dysregulated Snail-1 expression correlates with diminished E-cadherin and claudin levels and increased fibronectin and vimentin expression (11). Moreover, Snail-1 upregulates matrix metalloproteinase (MMP)-9, further enhancing tumor cell migration and invasion (11, 12). Numerous studies have underscored Snail-1’s impact on EMT-associated molecules, including E-cadherin, vimentin, MMP-9, and microRNAs like miR-34a, shaping cancer cells’ invasive, migratory, and metastatic potential (13–17).

In this study, we utilized siRNA-mediated silencing of Snail-1 in the EC-associated HEC-1A cell line to investigate its impact on the EMT process. We assessed the functional consequences of Snail-1 knockdown by evaluating changes in EMT-associated molecules, adaptor molecules in various pathways, cellular migration, and cytotoxic responses in siRNA-transfected cells.

## 2 Methods

### 2.1 Cell culture

HEK293 and MCF-7 cell lines were purchased from the Cell Bank of the Chinese Academy of Sciences (Shanghai, China). A549 and HEC-1A cell lines were obtained from the American Type Culture Collection (ATCC, Manassas, VA, USA). The EC-associated HEK293, HEC-1A, MCF-7, and A549 cell lines were selected for transfection and maintained in Roswell Park Memorial Institute (RPMI) 1640 culture medium (Sigma-Aldrich, St. Louis, MO, USA), supplemented with 10% fetal bovine serum (FBS; Sigma-Aldrich, St. Louis, MO, USA) and 1% penicillin/streptomycin antibiotics (Gibco Inc., Paisley, UK). Cultures were maintained at 37°C, 5% CO_2_, and 95% humidity under standard conditions. The medium was refreshed every 24 hours, and passaging occurred when cell confluency reached approximately 80–90%. The cells were detached using trypsin digestion for 5 minutes at 37°C.

### 2.2 siRNA transfection of HEC-1A cells

DNA oligonucleotides (siRNAs) targeting Snail-1 comprised three pooled siRNA duplex sequences shown in **Table 1**. A negative scrambled control siRNA (Santa Cruz Biotechnology, Inc) was also transfected into the control HEC-1A group. 2×10–^5^ cells per well were cultured in 6-well plates for transfection. After 18 hours, varying transfection reagents (HiPerfect^®^ Transfection Reagent, Qiagen, Hilden, Germany) and siRNA doses were added to the cells with at least 70% confluency. Cell harvesting occurred at 24-, 48-, and 72-hours post-transfection, followed by RNA and protein content isolation from the cells.

**Table 1:** Snail-1 siRNA sequences.

Strand	Sequences (3'-5')
Sense	GGACUUUGAUGAAGACCAUtt
Anti-sense	AUGGUCUUCAUCAAAGUCCtt
Sense	CACGAGGUGUGACUAACUAtt
Anti-sense	UAGUUAGUCACACCUCGUGtt
Sense	GCGAGCUGCAGGACUCUAAtt
Anti-sense	UUAGAGUCCUGCAGCUCGCtt

### 2.3 RNA extraction and cDNA synthesis

Total RNA extraction from HEK293, HEC-1A, MCF-7, and A549 cells was performed using Trizol (Qiagen, Germany) following the manufacturer’s instructions. The purity of the isolated RNA was assessed using a NanoDrop spectrophotometer (2000c, Thermo Fisher Scientific, USA) at 260 and 280 nm (260/280 ratio). The integrity of the extracted RNAs was evaluated using agarose gel electrophoresis. Subsequently, reverse transcription of the extracted RNAs was carried out to generate complementary DNA (cDNA) using the TAKARA cDNA Synthesis Kit (TAKARA, Japan) according to the manufacturer’s protocol. To assess the transcript level of miR-34a, the miScript II RT Kit (Cat No. 218161, Qiagen, Hilden, Germany) was utilized for reverse transcription of the extracted RNA to cDNA.

### 2.4 Quantitative Real-time-PCR

Quantitative Real-time PCR (qPCR) was conducted to detect mRNA and miRNA expressions using the RealQ Plus Master Mix Green High ROX (AMPLIQON, Odense M, Denmark) and the StepOne Plus Real-time PCR device (Applied Biosystems, Foster City, CA, USA). Primers for qPCR were obtained from Primer Bank (https://pga.mgh.harvard.edu/primerbank/, and the National Center for Biotechnology Information, as shown in **Table 2**). mRNA and miRNA transcript levels were normalized using the expression level of the corresponding housekeeping gene *Actin-β*. The qPCR reactions were prepared in a total volume of 20 µL, consisting of 10 µL of Master Mix, 0.5 µL of each forward and reverse primer (10 µM), 1 µL of diluted cDNA, and 8 µL of nuclease-free water. The cycling conditions included an initial denaturation step at 95°C for 3 minutes, followed by 40 cycles of 95°C for 15 seconds (denaturation), annealing at the specific temperatures of 56°C for *E-cadherin*, 61°C for *Snail-1*, 60°C for *MMP-9*, 60°C for *Notch1*, 58°C for *ERK*, 57°C for *AKT*, 55°C for *actin β*, and ~55–60°C for miRNAs for 30 seconds and extension at 72°C for 30 seconds. After 40 cycles, a final extension step was performed at 72°C for 5 minutes, followed by a melt curve analysis from 65°C to 95°C in 0.5°C increments. The qPCR instrument was set to high ROX mode to ensure proper signal normalization. The widely employed comparative CT method, as recommended by Schmittgen and Livak, was utilized to calculate the relative expression levels of target genes using the 2^-ΔΔCT^ formula (18).

**Table 2:** The primer sequences exerted in the real-time expression analysis in HEC-1A cells.

Gene name	Strand	Sequences
E-cadherin	Forward	5'-TCCATTTCTTGGTCTACG CC-3'
	Reverse	5'-CACCTTCAGCCAACCTGTTT-3'
Snail-1	Forward	5'-GGTTCTTCTGCGCTACTGCTG-3'
	Reverse	5'-GTCGTAGGGCTGCTGGAAGG-3'
MMP-9	Forward	5'-ATTTCTGCCAGGACCGCTTCTAC-3'
	Reverse	5'-ATCCGGCAAACTGGCTCCTTC-3'
Vimentin	Forward	5'-CAGGCAAAGCAGGAGTCCA-3'
	Reverse	5'-AAGTTCTCTTCCATTTCACGCA-3'
β-actin	Forward	5'-TCCCTGGAGAAGAGCTACG-3'
	Reverse	5'-GTAGTTTCGTGGATGCCACA-3'
Notch1	Forward	5'-CAGAGGCGTGGCAGACTAT-3'
	Reverse	5'-CGGCACTTGTACTCCGTCA-3'
AKT	Forward	5'-ACTGTCATCGAACGCACCTT-3'
	Reverse	5'-CTCCTCCTCCTCCTGCTTCT-3'
ERK	Forward	5'-TCCTTTGAGCCGTTTGGAGG-3'
	Reverse	5'-TACATACTGCCGCAGGTCAC-3'
miR-34a	Target sequence	5'-UGGCAGUGUCUUAGCUGGUUGU-3'

### 2.5 MTT assay

The methyl-thiazol-tetrazolium (MTT) assays were conducted to assess cell viability following treatment of HEC-1A cells with different doses of Snail-1 specific siRNA (20, 40, 60, and 80 pmol/mL). HEC-1A cells (5×10^3^) were seeded in triplicate in 96-well plates, with each well containing 100 μl RPMI 1640 medium. Transfection was performed as described previously. Subsequently, cells from both control and transfected groups were cultured for 24, 48, and 72 hours. For the MTT assay, 100 µl of MTT reagent (Sigma, Germany) at a concentration of 0.0005 g/ml in PBS was added to each well, followed by a 4-hour incubation period. Afterward, 100 µl of dimethyl sulfoxide (DMSO) was added to halt formazan crystal production, and plates were incubated for an additional 30 minutes on a shaker at room temperature. Finally, each well’s optical density (OD) was measured at 570 nm wavelength using an ELISA reader (Tecan Spectra, Austria).

### 2.6 Western Blot analysis

Protein extraction from HEC-1A cells was performed using RIPA buffer (25 mM Tris HCl pH 7.6, 150 mM NaCl, 1% NP-40, 1% sodium deoxycholate, 0.1% SDS). Subsequently, 100 μg of the extracted total protein was subjected to electrophoresis on a 12.5% Sodium dodecyl sulfate-polyacrylamide gel (SDS-PAGE), followed by electroblotting onto Polyvinylidene fluoride (PVDF) membranes. To block the membranes, 3% bovine serum albumin (BSA) in TBST solution (1× Tris-Buffered Saline, 0.1% Tween-20) was added and incubated at room temperature overnight. Primary Rabbit polyclonal antibodies were utilized for detecting protein levels of Snail-1 (1:500, sc-28199, Santa Cruz Biotechnology), MMP-9 (ab283575, Abcam), E-cadherin (ab40772, Abcam), vimentin (ab92547, Abcam), Notch1 (ab52627, Abcam) and β-actin (1:3000, monoclonal antibody, Abcam) as the housekeeping protein. Following washing, the membranes were incubated with horseradish peroxidase (HRP)-conjugated goat anti-rabbit secondary polyclonal antibody (1:3000, Santa Cruz Biotechnology). Protein levels were evaluated using the electrochemiluminescence (ECL) kit (Roche Diagnostics GmbH). Quantification of protein levels was performed using NIH ImageJ 1.63 software.

### 2.7 Apoptosis evaluation by flow cytometry

Flow cytometric apoptosis analysis was conducted to evaluate the apoptotic potential of the Snail-1-specific siRNA. Annexin-V-FLUOS staining kit, supplied by Roche Diagnostics, was employed according to the manufacturer’s instructions. This method distinguishes between apoptotic and necrotic cells with precision. The experimental procedure involved seeding 2 × 10^5^ HEC-1A cells, allowing them to increase for 24 hours. Subsequently, the cells were treated with Snail-1-specific siRNA at a concentration of 60 pmol/mL for 72 hours at 37°C. Following treatment, 1 × 10^6^ cells underwent PBS wash and were centrifuged at 200 g for 5 minutes. The resulting cell pellet was resuspended in 100 μL of labeling solution containing Annexin-V-FLUOS labeling reagent and propidium iodide (PI) solution. After a 15-minute incubation at 25°C, the cell suspensions were analyzed in triplicate using a BD Bioscience FACScaliber flow cytometer (BD, USA).

### 2.8 Wound healing assays

A wound healing assay was conducted to assess the metastatic and migratory potential of HEC-1A cells following transfection with Snail-1 specific siRNA. This assay evaluates the ability of transfected cells to close a gap area created in a cell monolayer. Initially, 10×10^5^ HEC-1A cells were seeded per well in 24-well plates. Once cells reached 90% confluency, a scratch was carefully made across the cell monolayer using a 200-µL sterile pipette tip, creating a linear gap region. Subsequently, the plate surface was washed with PBS to remove cell debris. HEC-1A cells were then transfected with 80 pmol of Snail-1-specific siRNA. Experiments were conducted in triplicate. Images of the plates were captured at 0-, 24-, 48-, and 72-hours post-scratching using light microscopy. The number of migrated cells was quantified using NIH ImageJ 1.63 software. This analysis provides insights into the migratory capacity of HEC-1A cells following Snail-1 siRNA transfection.

### 2.9 Statistical analysis

Statistical analysis and graph plotting were performed using GraphPad Prism v.9 software (GraphPad Software, La Jolla, California, USA). Depending on the data distribution, either one-way ANOVA or the non-parametric Kruskal–Wallis test was employed to assess group differences. Data were presented as mean ± standard deviation (SD) from three independent experiments. Statistical significance was considered at P values less than 0.05.

## 3 Results

### 3.1 mRNA expression of Snail-1 in various cell lines

**Figure 1:**
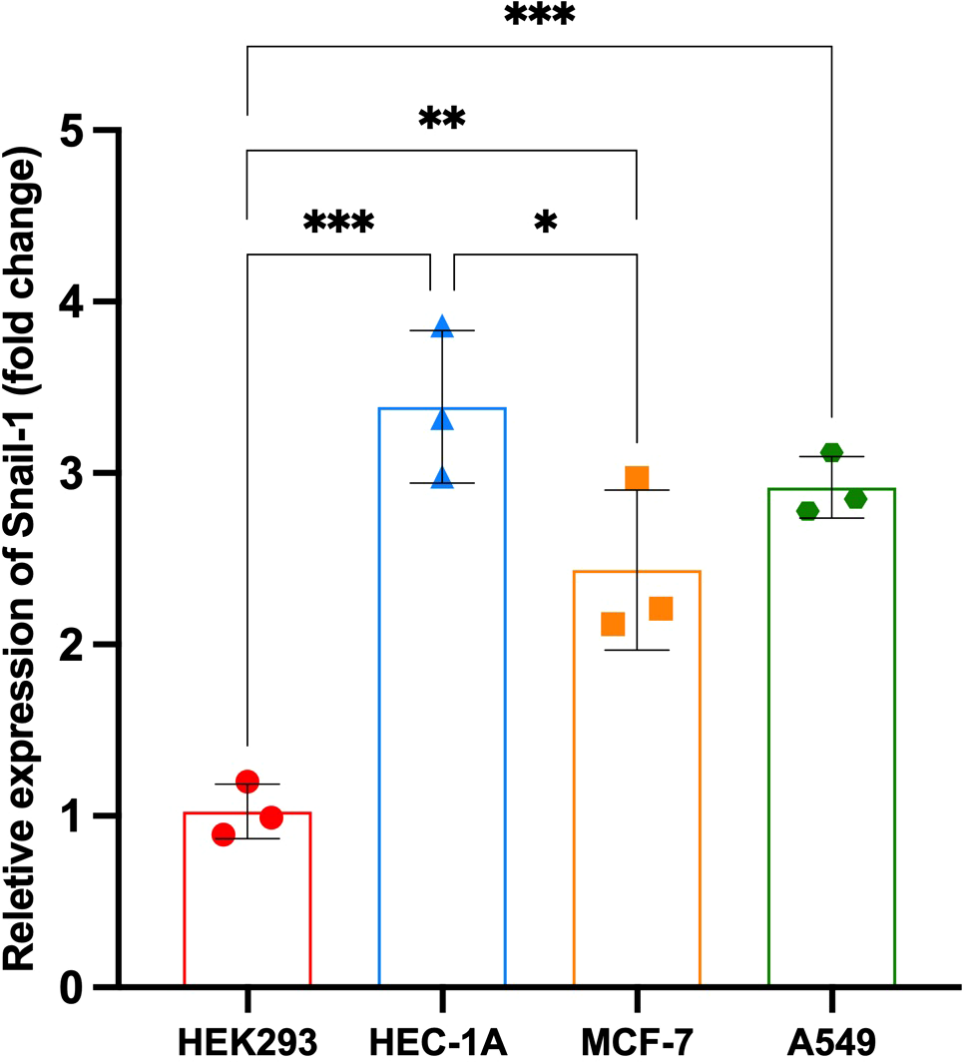
Relative expression of Snail-1 across different cell lines. Bar graph depicting fold change in *Snail-1* expression in HEK293, HEC-1A, MCF-7, and A549 cell lines. Data are presented as mean ± SD from three independent experiments. Statistical comparisons were made using one-way ANOVA followed by Tukey’s multiple comparisons test. ***, **, and * indicate *P* < 0.001, *P* < 0.01, and *P* < 0.05, respectively. HEC-1A cells displayed the highest *Snail-1* expression, significantly greater than HEK293 and MCF-7. A549 cells also showed significantly increased expression compared to HEK293. ns, not significant.

The relative expression of *Snail-1* was significantly different among the tested cell lines (HEK293, HEC-1A, MCF-7, and A549). HEK293 cells exhibited the lowest expression levels, with a mean fold change of approximately 1.0, serving as the baseline. In contrast, *Snail-1* expression was markedly upregulated in HEC-1A cells (**P = 0.0001 vs. HEK293), A549 cells (**P = 0.0007), and MCF-7 cells (*P = 0.0045). Moreover, HEC-1A cells showed significantly higher *Snail-1* expression than MCF-7 cells (P = 0.038), while no statistically significant differences were observed between HEC-1A and A549 or between MCF-7 and A549 (P > 0.05). These findings suggest that *Snail-1* is differentially expressed across epithelial cancer cell lines, with the highest expression in HEC-1A, implicating a potentially more mesenchymal phenotype (**Figure 1**).

### 3.2 mRNA expression of Snail-1

**Figure 2:**
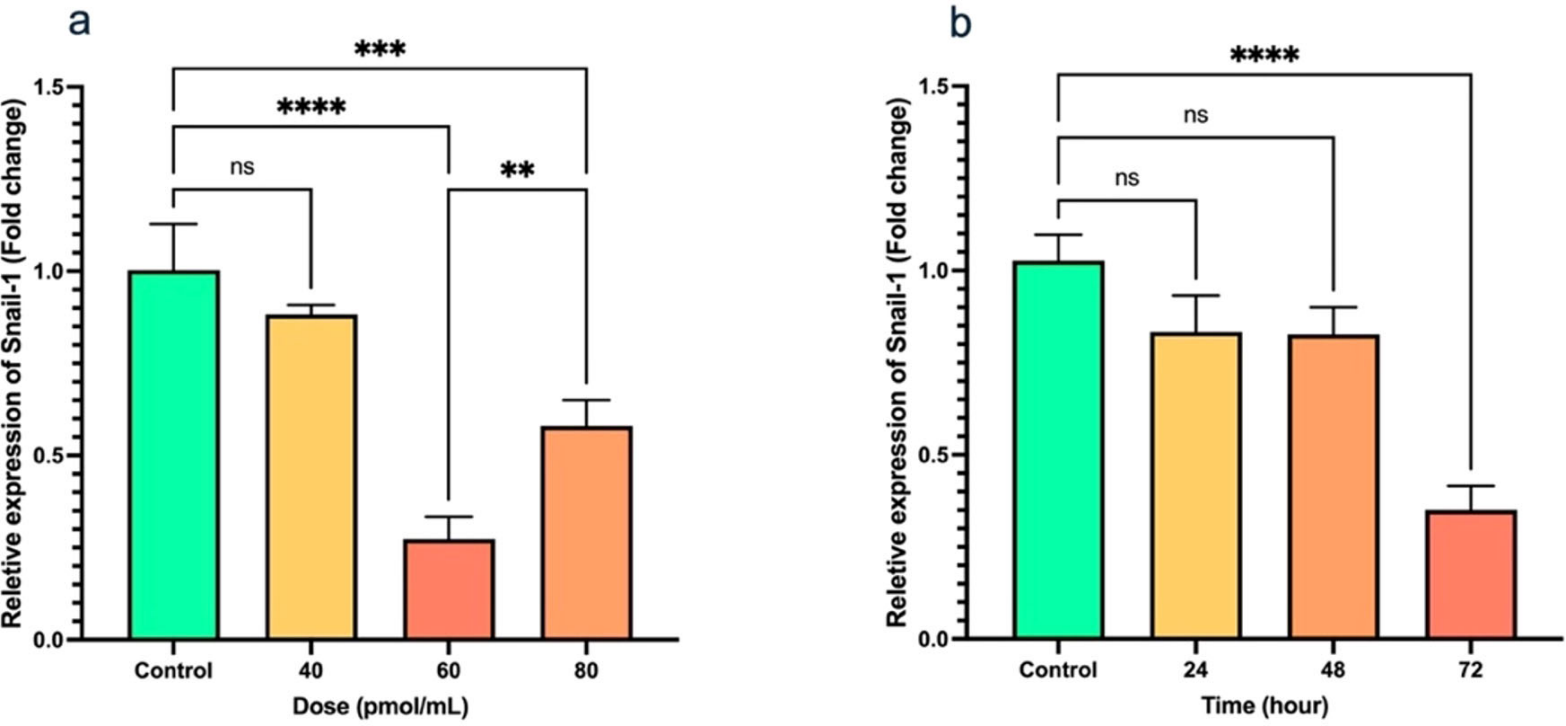
Transfection of HEC-1A cells by Snail-1 specific siRNA and aftermath expression of Snail-1. **(a)** Snail-1 mRNA expression following 48 hrs transfection of HEC-1A cells with three doses of 40, 60, and 80 pmol of Snail-1 specific siRNA. **(b)** mRNA expression of Snail-1 in HEC-1A cells after 24, 48, and 72 hrs from transfection of HEC-1A cells with 60 pmol of Snail-1 specific siRNA. The experiments were done in triplicate (Data are represented as mean ± SD; ** indicates *P* < 0.01, *** indicates *P* < 0.001, and ****P < 0.0001), ns, not significant.

The findings showed that upon treatment with 40, 60, and 80 pmol/mL doses of Snail-1 specific siRNA, a remarkable downregulation in Snail-1 mRNA expression was observed at 60 pmol/mL (P < 0.0001) in Snail-1 transfected HEC-1A cells compared with other doses and untreated control cells (**Figure 2a**). In addition, following transfection of HEC-1A cells with the effective dose (60 pmol/mL) of Snail-1 specific siRNA, a discernible decrease in *Snail-1* mRNA expression was observed at 24 hrs, 48 hrs, and 72 hrs post-transfection. However, statistical significance in the downregulation of Snail-1 mRNA expression was solely evident at 72 hrs (P < 0.0001) when compared to the control group (**Figure 2b**). These findings emphasize the efficacy of Snail-1 specific siRNA in modulating Snail-1 mRNA expression levels in HEC-1A cells in 60 pmol/mL at 72 hrs as effective dose and time, respectively.

### 3.3 Snail-1 protein level

**Figure 3:**
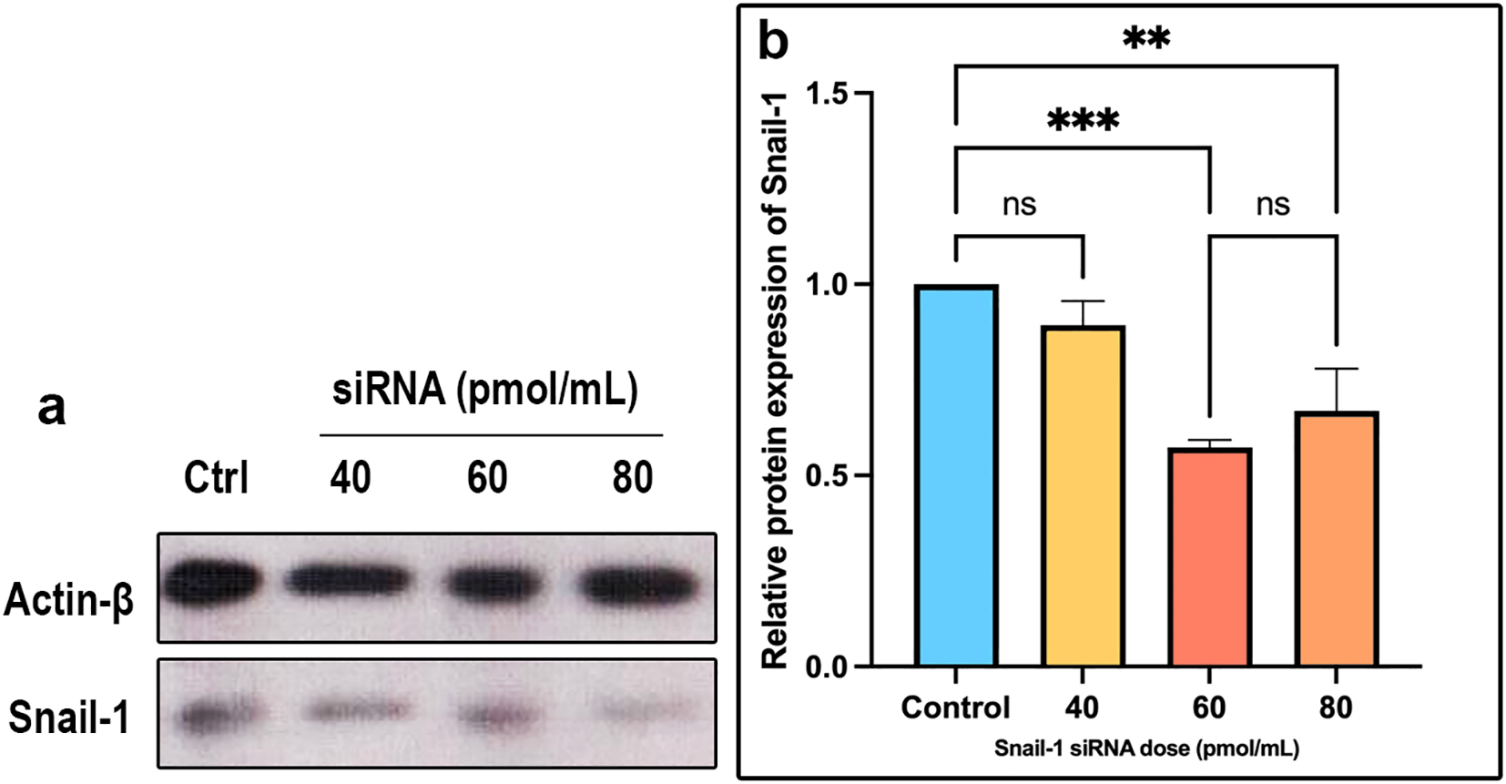
Expression of Snail-1 protein in HEC-1A cells transfected by Snail-1 specific siRNA. HEC-1A cells were transfected with three doses of 40 pmol, 60 pmol, and 80 pmol of Snail-1 specific siRNA. **(a)** The expression level of each band was identified via densitometry, and the color density of each band was normalized to the β-actin protein level. **(b)** Protein expression of Snail-1 was significantly decreased at 60 and 80 pmol of Snail-1 specific siRNA. (Data are represented as mean ± SD; ** indicates *P* < 0.01, and *** indicates *P* < 0.001). ns, not significant.

Following transfection of HEC-1A cells with Snail-1 specific siRNA, the intensity of the protein bands indicated a notable downregulation in Snail-1 protein expression. Statistical analysis revealed significant decreases at 60 pmol/mL (P= 0.0002) and 80 pmol/mL (P= 0.001) concentrations compared to the negative control group after 72 hours. Interestingly, the efficacy peaked at the 60 pmol/mL dose, surpassing 40 and 80 pmol/mL concentrations. These results underscore the potential of Snail-1-specific siRNA as a promising therapeutic avenue for targeted gene silencing in HEC-1A cells (**Figures 3a, b**).

### 3.4 Induction of cytotoxicity with Snail-1 specific siRNA

**Figure 4:**
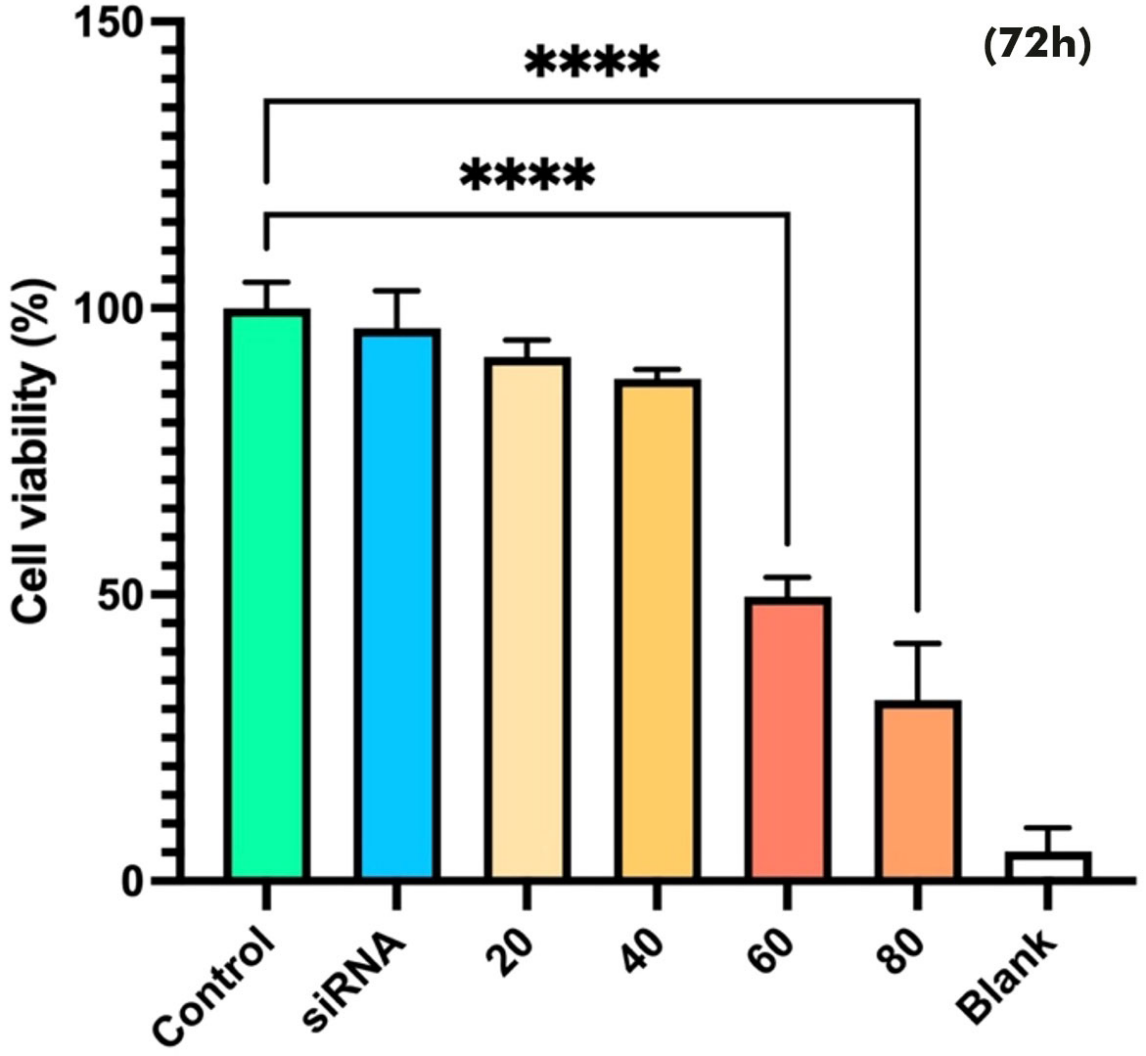
Effect of Snail-1 siRNA on HEC-1A cell line. At 72 h after transfection with snail-1 siRNA (20, 40, 60, and 80 pmol/mL), the cytotoxicity of treatments was determined by MTT assay as described in the methods section. (Data are represented as mean ± SD; **** indicates *P* < 0.0001).

The effect of Snail-1 downregulation on HEC-1A cells was also investigated. Cell viability was assessed using the MTT assay at 24, 48, and 72 hours after treatment. However, only the 72-hour results are presented, as they showed statistically significant and consistent changes in cell viability. At 24 and 48 hours, the results were not sufficiently distinct or reproducible to draw reliable conclusions. As shown in **Figure 4**, monotherapy with Snail1-specific siRNA induced cytotoxicity. The results of the MTT assay showed that 60 and 80 pmol/mL Snail-1 siRNA group significantly decreased the percentage of cell viability, compared with the control group at 72 hrs (P < 0.0001).

### 3.5 Induction of apoptosis with Snail-1 specific siRNA

**Figure 5:**
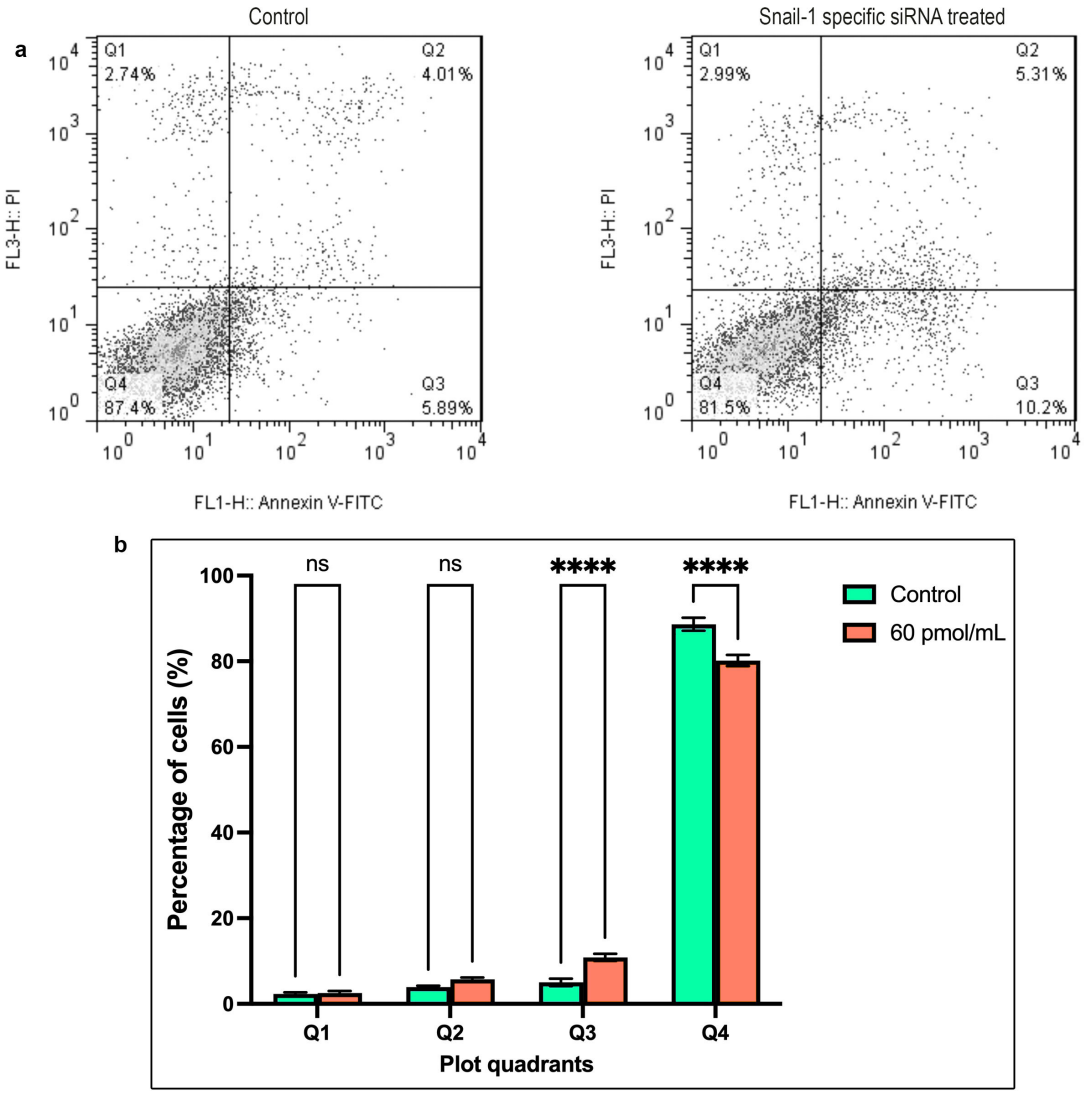
The apoptosis rate of HEC-1A cells after transfection with 60 pmol/mL as the effective dose of Snail-1 specific siRNA. Apoptosis of HEC-1A cells was determined by Annexin-V/PI and flow cytometry at 72 hrs after transfection. **(a)** The percentage of necrotic (Q1), late apoptotic (Q2), early apoptotic (Q3), and normal cells (Q4) in untreated and treated HEC-1A cells. **(b)** The differences between the percentage of cell death in untreated and treated cells in each quadrant are demonstrated. The results are presented as mean ± SD (n=3), and **** indicates *P*< 0.0001, ns, not significant.

Annexin V/PI staining was conducted on HEC-1A cells to evaluate the effect of a potent Snail-1-specific siRNA (60 pmol/mL) over a 24, 48, and 72-hour time frame on apoptosis. As depicted in **Figures 5a, b**, cells transfected with the siRNA demonstrated a significantly elevated percentage of early apoptosis compared to the control (*P* < 0.0001). These observations indicate a pronounced augmentation in cell apoptosis upon treatment with 60 pmol/mL of Snail-1-specific siRNA for 72 hours, evidenced by a statistically significant difference (**Figure 5**).

### 3.6 Expression of target genes and miR-34a

**Figure 6:**
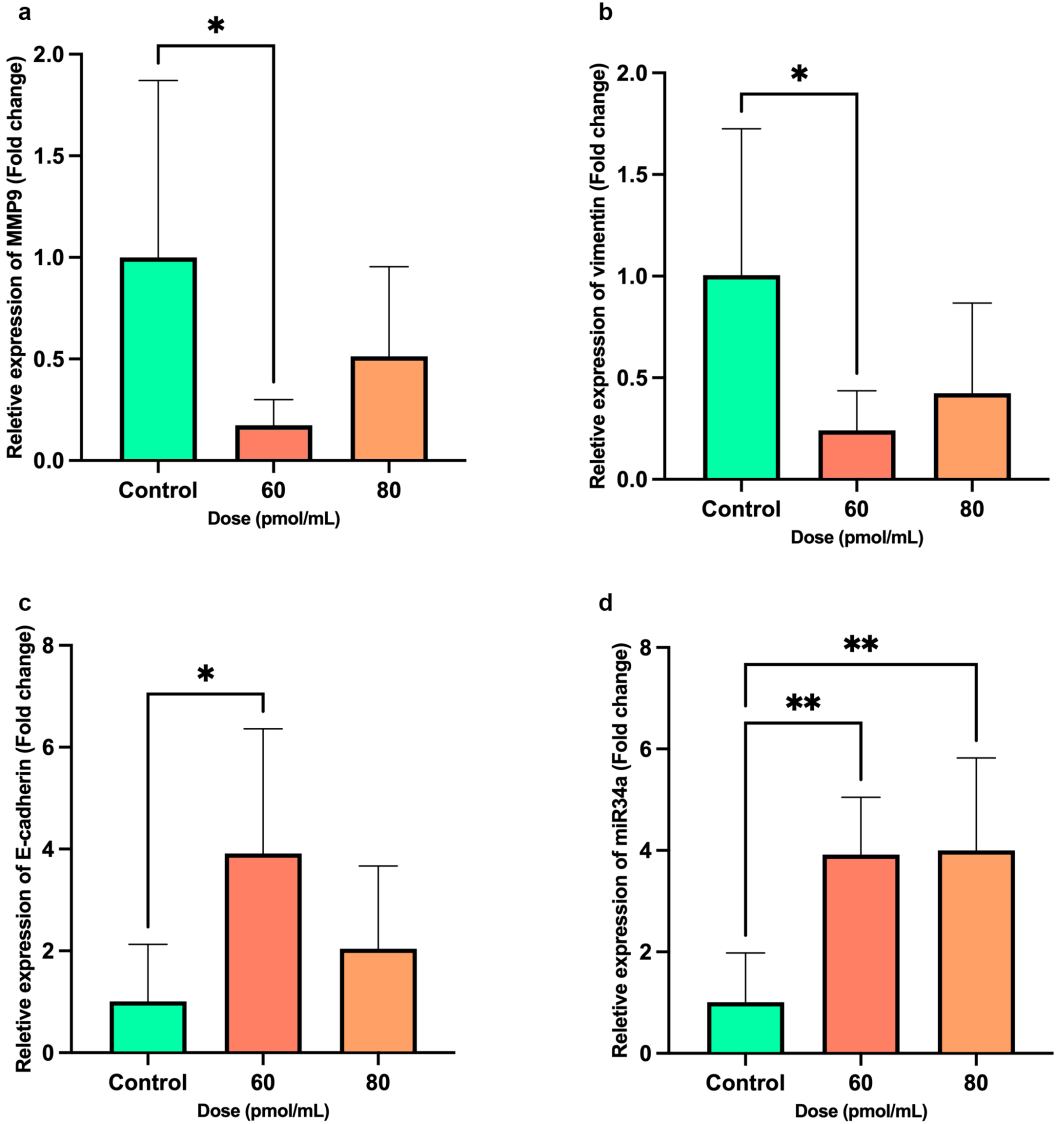
Bar graphs show the expression levels of metastatic-related genes, including *MMP9*, *Vimentin*, *E-cadherin*, and *miR-34a* after transfection of HEC-1A cells by Snail-1 specific siRNA after 72 hrs. mRNA levels of **(a)** *MMP-9*, **(b)** *Vimentin*, **(c)** *E-cadherin*, and **(d)** *miR-34a* were evaluated after transfection of HEC-1A cells by three doses of 60 and 80 pmol/mL as effective doses of Snail-1 specific siRNA compared with the untreated cells as control using quantitative Real-time PCR. The experiments were done in triplicate (Data are represented as mean ± SD; * indicates *P*<0.05, ** *P*<0.01).

Transfection with Snail-1 specific siRNA led to a substantially reduced *MMP-9* mRNA expression at 60 pmol of siRNA (*P* = 0.042) compared with the control group (**Figure 6a**). While transfection with Snail-1 specific siRNA showed a trend toward downregulating *vimentin* expression in HEC-1A cells following treatment with 60 pmol/mL, this effect did reach statistical significance (*P* = 0.034) (**Figure 6b**). Conversely, transfection with Snail-1 specific siRNA at 60 pmol/mL significantly increased *E-cadherin* expression (*P* = 0.026) (**Figure 6c**). A remarkable elevation in *miR-34a* expression was observed in HEC-1A cells upon transfection with 60 (*P* = 0.0039) and 80 pmol/mL (*P* = 0.0032) of Snail-1 specific siRNA (**Figure 6d**).

**Figure 7:**
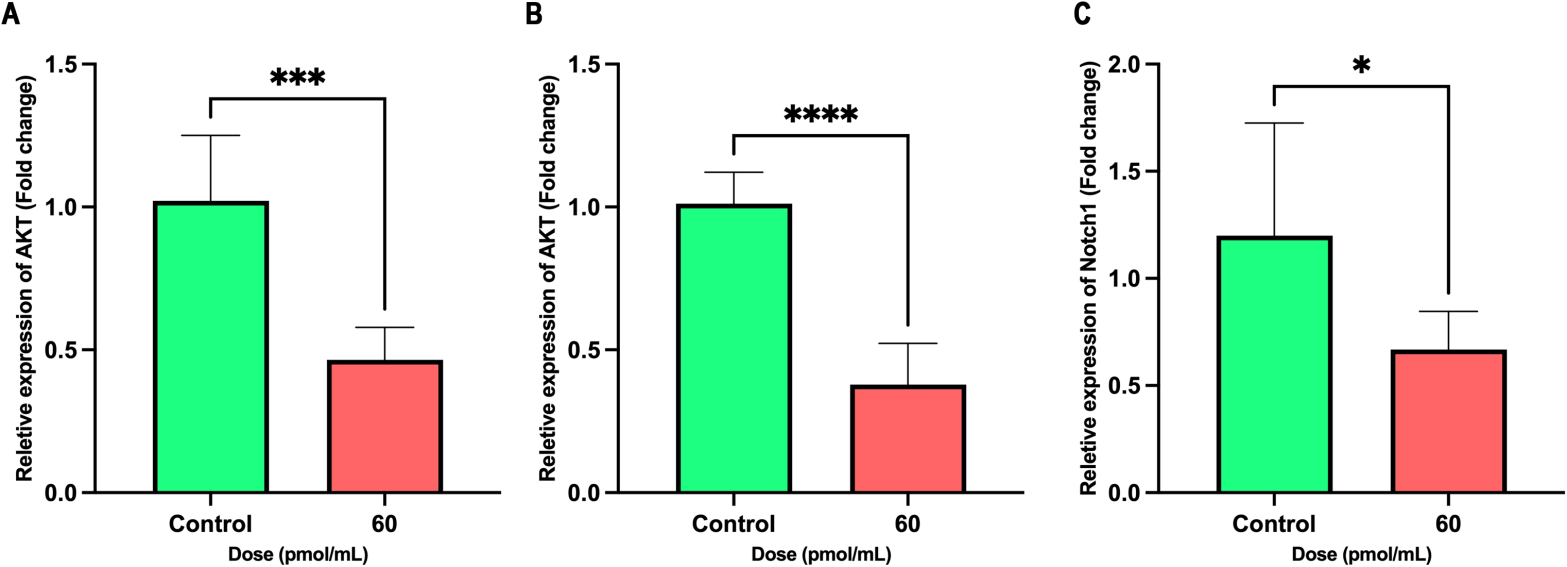
Inhibitory effects of siRNA treatment (60 pmol/mL) on *AKT*, *ERK*, and *Notch1* expression. Bar graphs show the relative mRNA expression of **(A)** *AKT*, **(B)** *ERK*, and **(C)** *Notch1* in control (green) and treated (red) groups. Significant decreases were observed in all three genes upon treatment. Data are presented as mean ± SD (n = 6). Unpaired two-tailed t-tests were used for statistical analysis. P < 0.05 (*), P < 0.001 (***), and P < 0.0001 (****) indicate significance levels.

Treatment with the siRNA at a concentration of 60 pmol/mL significantly reduced the mRNA expression of *AKT*, *ERK*, and *Notch1* in cancer cells. As illustrated in **Figure 7**, *AKT* expression was markedly suppressed in the treated group compared to controls (P < 0.0001), indicating potent inhibition of this prosurvival pathway. A significant downregulation of *ERK* expression was also observed (P = 0.0003), supporting an effect on μitogen-activated protein kinases (MAPKs) signaling. Moreover, a moderate but statistically significant reduction in *Notch1* expression was detected (P = 0.0407). These findings suggest that the siRNA exerts its effects by concurrently targeting multiple signaling pathways associated with proliferation and differentiation.

### 3.7 Expression of Snail-1-related proteins

**Figure 8:**
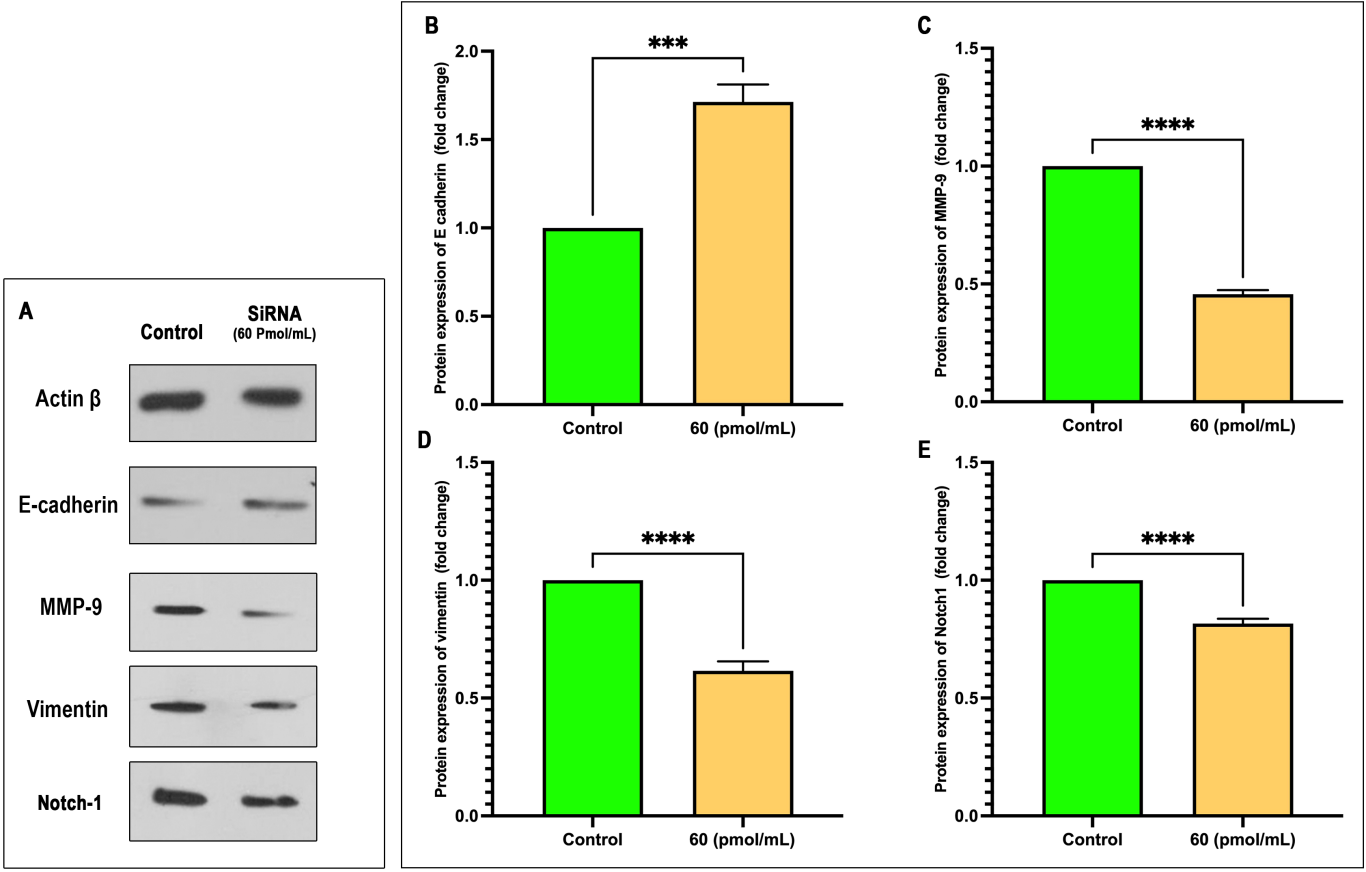
siRNA treatment at 60 pmol/mL modulates EMT marker expression. **(A)** Representative Western blot images of Actin β, E-cadherin, MMP-9, Vimentin, and Notch1 following siRNA treatment versus control. **(B–E)** Quantitative analysis of protein expression (fold change relative to control) for E-cadherin **(B)**, MMP-9 **(C)**, Vimentin **(D)**, and Notch1 **(E)**. siRNA significantly increased E-cadherin expression while reducing MMP-9, Vimentin, and Notch1 levels. Data are presented as mean ± SEM (n = 3). Statistical analysis was performed using unpaired two-tailed t-tests: ***p < 0.001, ****p < 0.0001.

Cell treatment by siRNA at 60 pmol/mL significantly modulated the expression of key EMT markers. Western blot analysis (**Figure 8A**) and densitometric quantification (**Figures 8B–E**) demonstrated a marked upregulation of E-cadherin in the siRNA group compared to control (mean fold change: 1.714 vs. 1.000; P = 0.0002; 95% CI: 0.5556 to 0.8717), indicating a shift toward an epithelial phenotype. Conversely, mesenchymal markers MMP-9, Vimentin, and Notch1 were significantly downregulated following siRNA treatment. MMP-9 levels decreased by over 50% (mean: 0.4558 vs. 1.000; P < 0.0001; 95% CI: –0.5718 to –0.5167), with an exceptionally high effect size (R² = 0.9987). Similarly, Vimentin expression dropped significantly (mean: 0.6155; P < 0.0001; 95% CI: –0.4493 to –0.3197), and Notch1 was modestly but significantly reduced (mean: 0.8164; P < 0.0001; 95% CI: –0.2162 to –0.1511). These results collectively suggest that siRNA effectively reverses EMT, potentially by downregulating Notch1 signaling and inhibiting mesenchymal marker expression.

### 3.8 Migration of HEC-1A cells

**Figure 9:**
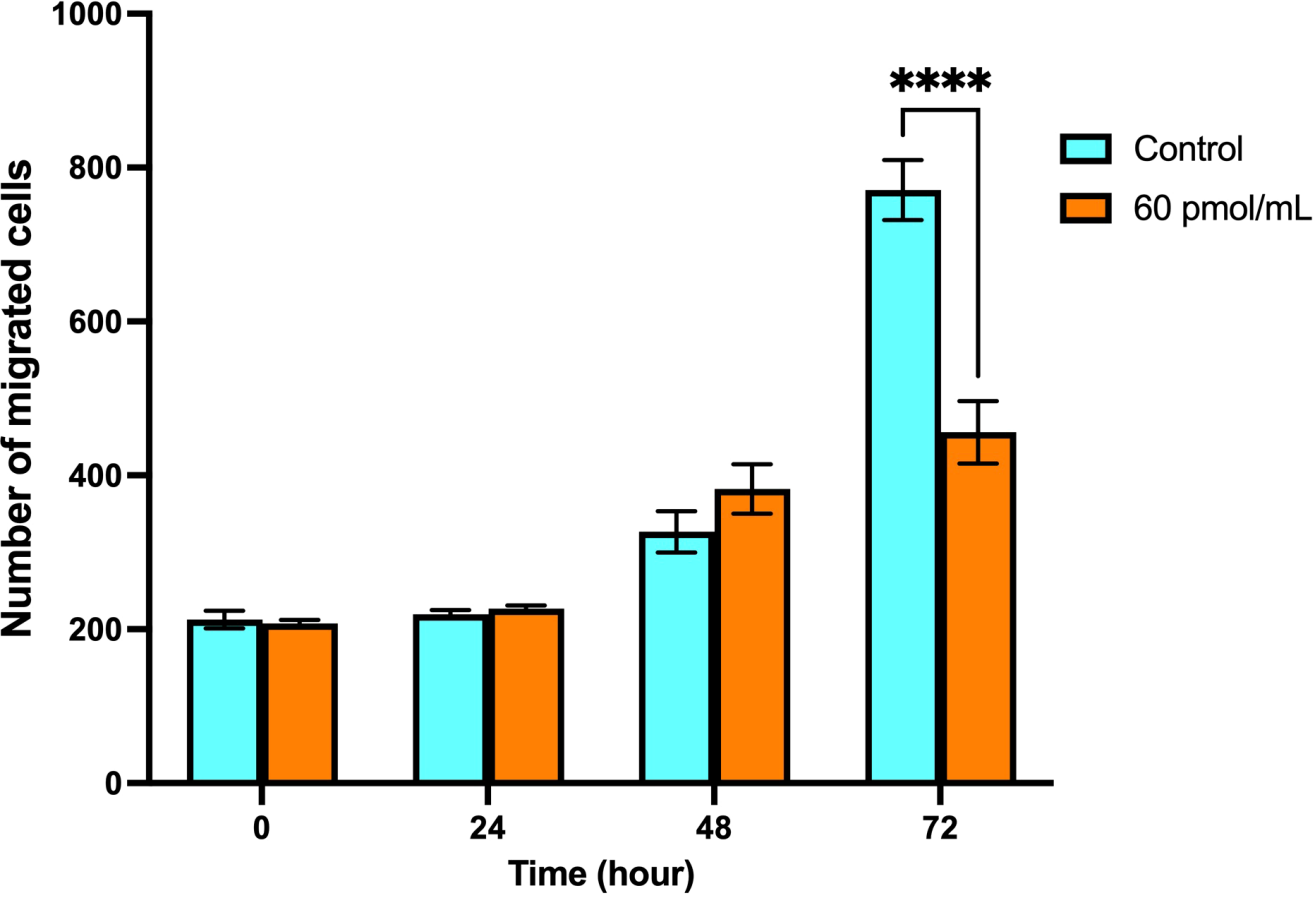
Migration of HEC-1A cells after transfection with Snail-1 specific siRNA since 0 hrs, 24 hrs, 48 hrs, and 72 hrs. A monolayer of HEC-1A cells seeded on wells was transfected by Snail-1 specific siRNA. A scratch was generated on the plate surface, and the filling of gaps was evaluated after 0 hrs, 24 hrs, 48 hrs, and 72 hrs (Data are presented as means ± SD; **** indicates *P*<0.0001).

According to the wound healing assay, it was observed that transfection of Snail-1 specific siRNA resulted in a significant reduction in the number of migrated HEC-1A cells to the scratched region at 72 hrs (*P*< 0.0001), compared with the control group (**Figure 9**).

## 4 Discussion

The phenomenon known as EMT, characterized by converting epithelial cells into a mesenchymal phenotype endowed with migratory capabilities, plays a crucial role in the progression of malignancies (19). This intricate biological process is central to tumor advancement and metastatic dissemination, profoundly impacting various cancerous behaviors such as invasion, migration, and metastasis (20–22). Consequently, there is a significant interest in investigating the molecular pathways governing invasion and metastasis, particularly within the context of EMT in the EC (23).

Our findings demonstrate differential *Snail-1* expression across the tested cell lines (HEK293, HEC-1A, MCF-7, and A549), with HEK293 showing the lowest expression as the baseline. Notably, *Snail-1* was significantly upregulated in HEC-1A, A549, and MCF-7 cells, with HEC-1A exhibiting the highest expression, suggesting a potential association with a more mesenchymal phenotype. While HEC-1A had significantly higher *Snail-1* levels than MCF-7, no significant differences were observed between HEC-1A and A549 or MCF-7 and A549. These results indicate that *Snail-1* expression varies across epithelial cancer cell lines, with the highest levels observed in HEC-1A, pointing to its potential role in promoting EMT and cancer progression (24).

Numerous studies have highlighted the pivotal role of Slug/Snail zinc-finger proteins in driving the tumorigenic behaviors of malignant cells, primarily through the attenuation of epithelial cell adhesive properties (25–27). Snail family proteins, particularly Snail-1, are promising targets for developing targeted therapeutic interventions (11, 28). Modifying these proteins presents a potential avenue for disrupting the EMT process and impeding tumor progression in EC. Therefore, elucidating the intricate mechanisms by which Slug/Snail proteins contribute to EMT in EC holds significant therapeutic implications for managing this aggressive disease (29). Further exploring these molecular pathways may unveil novel therapeutic targets and strategies for combating EC progression and metastasis.

Snail-1 is a key transcription factor that drives EMT in various cancers, including EC, breast, lung, and ovarian (28, 30). In EC, Snail-1 promotes tumor progression, invasion, and metastasis, with a unique sensitivity to hormonal regulation, particularly estrogen and progesterone signaling (31). Despite Snail-1 involvement in EMT and therapy resistance, it appears Snail-1’s function in EC and breast cancer is distinct due to its hormonal influence and specific metastatic behavior, underscoring the need for targeted research to explore its unique molecular mechanisms and therapeutic implications (32).

Gene silencing via RNA interference (RNAi) has emerged as a promising personalized strategy for treating malignancies, including EC. siRNAs are potent effector molecules capable of silencing critical genes implicated in cancer pathogenesis (33). Despite the array of therapeutic modalities available for EC treatment, including hormonal therapy, surgery, chemotherapy, immunotherapy, radiation therapy, or combination approaches, their efficacy remains limited (34).

In light of this clinical challenge, our study aims to investigate the therapeutic potential of Snail-1 silencing using siRNA to mitigate EC’s invasive and metastatic characteristics *in vitro*. We hypothesize that targeted inhibition of Snail-1 expression could disrupt the underlying molecular pathways associated with EMT and consequently attenuate the aggressive behavior of EC cells (31). By elucidating the effects of Snail-1 silencing on EC cell behavior, our research endeavors to contribute valuable insights into developing novel therapeutic strategies to improve the clinical outcomes for EC patients.

The pivotal role of Snail-1 in orchestrating the metastatic phenotype of tumor cells has been well-documented (11). *In vitro* studies have provided compelling evidence demonstrating the indispensable role of Snail in facilitating tumor cell metastasis to lymph nodes (35). Moreover, elevated mRNA levels of Snail have been observed in metastatic lesions derived from ovarian cancer, underscoring its significance in cancer progression (36). Immunohistochemical analyses have further revealed the nuclear expression of Snail and Slug in a subset of EC tumors, with nuclear Snail expression indicative of EMT being significantly associated with aggressive clinicopathological features and poorer patient prognosis (37).

Furthermore, investigations into the regulatory mechanisms governed by Snail have unveiled its ability to modulate the expression of critical EMT-associated molecules such as E-cadherin, Notch1, vimentin, and MMP-9 in various cancer cell types, including gliomas (38). Consistent with these findings, our study hypothesizes that targeting Snail-1 expression could mitigate the metastatic behaviors of EC cells. Through targeted silencing of Snail-1 in HEC-1A cells, we observed a significant modulation in the expression levels of some EMT-related molecules, characterized by the downregulation of MMP-9, Notch1, and vimentin and concomitant upregulation of E-cadherin at mRNA and protein levels. These findings indicate the therapeutic potential of Snail-1 inhibition to attenuate EC’s invasive and metastatic features, providing a rationale for further exploration of Snail-targeted interventions in EC management.

While previous research has assessed Snail-1 expression levels in EC (37), studies still need to investigate the impact of Snail-1 knockdown on apoptosis and migration of EC tumor cells. Nevertheless, existing literature has suggested a link between Snail-1 silencing and promoting apoptosis in various tumor types (15, 39, 40). In line with these observations, our current study sought to elucidate the effects of Snail-1 knockdown on apoptotic pathways and cell migration in EC.

Our investigation employed Snail-1-specific siRNA transfection to modulate Snail-1 expression in HEC-1A cells. Remarkably, our findings revealed a significant increase in apoptosis following Snail-1 knockdown, highlighting a potential mechanism through which Snail-1 inhibition may exert its anti-tumorigenic effects in EC (41). This observation contributes to our understanding of the molecular mechanisms underlying EC progression. It emphasizes the therapeutic potential of targeting Snail-1 to promote tumor cell apoptosis and impede tumor progression.

It has been revealed that the expression of vimentin and fibronectin as the mesenchymal markers is decreased during EMT (42). In contrast, the expression of epithelial markers like mucin-1 and E-cadherin is increased (43). It has been reported that Snail knockdown reduced vimentin expression in breast cancer cell lines (35). Moreover, E-cadherin expression has been demonstrated to be decreased in EC, which is associated with EMT (37). Our experiments indicated that Snail-1 silencing resulted in the upregulation of E-cadherin and downregulation of *vimentin* and *MMP-9* in HEC-1A cells, which was associated with a marked decrease in cell migration and metastasis.

On the other hand, protease enzymes participate in the development of EMT by degrading the extracellular matrix, facilitating the migration and metastasis of malignant cells (44). MMPs have been reported to play a role in increasing the migration and metastasis of malignant cells (45–47). Invasion of hepatoma cells has been established to be under the impression of Snail function using MMP upregulation (48). Alternately, the generation of MMP-9 and vimentin was increased by Snail in the glioma cell lines (38). Our experiments revealed that silencing of Snail-1 downregulates *MMP-9* as a metastasis-related gene and decreases the number of migrated cells.

miR-34a has been implicated as a tumor suppressor in the pathogenesis of various malignancies (49, 50) and EC (51, 52). miR-34a was reported to be downregulated in EC tissues and negatively correlated with Notch1 expression. Moreover, miR-34a can suppress the proliferation, migration, invasion, and EMT-associated phenotypes via downregulating the Notch1 expression in EC cells. Additionally, upregulation of miR-34a repressed the tumor growth in nude mice (53). Underexpression of miR-34a was reported in the esophageal squamous-cell carcinomas. However, the upregulation of miR-34a culminated in the increased apoptosis of cancer cells while downregulating MMP-2 and MMP-9 expression, inhibiting invasiveness and migration of cancer cells (50). Our findings revealed that transfection of HEC-1A cells by Snail-1 specific siRNA can upregulate miR-34a and downregulate *MMP-9*, decreasing migration of HEC-1A cells.

Snail-1 exerts its regulatory functions through various signaling pathways, including TGF-β, Wnt/β-catenin, and Notch, which are critical in the induction of EMT, maintenance of stemness, and resistance to apoptosis (28). Nevertheless, the precise mechanistic interactions between Snail-1 and these signaling cascades in EC have yet to be elucidated fully. Treatment with siRNA at a concentration of 60 pmol/mL significantly decreased the mRNA expression of *AKT, ERK*, and *Notch1* in cancer cells (54). Notably, AKT expression was substantially suppressed in the treated group, indicating a potent inhibition of this prosurvival pathway. Significant downregulation of *ERK* expression was also observed, suggesting an effect on the MAPK signaling pathway. Additionally, *Notch1* expression was moderately but significantly reduced (55). These findings imply that siRNA effectively targets multiple signaling pathways involved in cellular proliferation and differentiation, which may contribute to its potential therapeutic effects in cancer treatment.

Taking all the findings together, this was the first investigation, to the best of our knowledge, to evaluate the role of *Snail-1* silencing by siRNA in impressing the EMT of EC HEC-1A cells. *Snail-1* specific siRNA reduced the expression of *Snail-1* at mRNA level in EC HEC-1A cells. Moreover, the apoptosis rate of HEC-1A cells was increased. Also, the migratory potential of HEC-1A cells was decreased upon transfection, alongside the downregulation of MMP-9, Notch1, and vimentin *and* the upregulation of E-cadherin at mRNA and protein levels. Accordingly, silencing *Snail-1* by specific siRNA suggests a potential therapeutic strategy for EC therapy. However, it needs further studies on other cancer cell lines and animal models.

Future research should focus on elucidating the precise molecular pathways through which Snail-1 contributes to EC progression and therapy resistance. Advanced omics technologies, including transcriptomics and proteomics, could help identify novel regulatory networks involving Snail-1. Additionally, *in vivo* studies and patient-derived models will be essential to validate the translational relevance of our findings. Investigating potential therapeutic strategies to target Snail-1, such as small-molecule inhibitors or RNA-based therapeutics, may pave the way for novel treatment approaches. Integrating Snail-1 research with immunotherapy and precision medicine strategies could lead to more effective and personalized interventions for EC patients.

## Data Availability

The datasets presented in this study can be found in online repositories. The names of the repository/repositories and accession number(s) can be found in the article/**Supplementary Material**.
