# Supplementary figures and images for: Unveiling the intricacies: small interfering RNA targeting Snail-1 unravels dynamics in endometrial carcinoma cell behavior
Source: Front Oncol. 2025 Jun 12;15:1567493. doi: 10.3389/fonc.2025.1567493 (PMC12197919; doi:10.3389/fonc.2025.1567493)

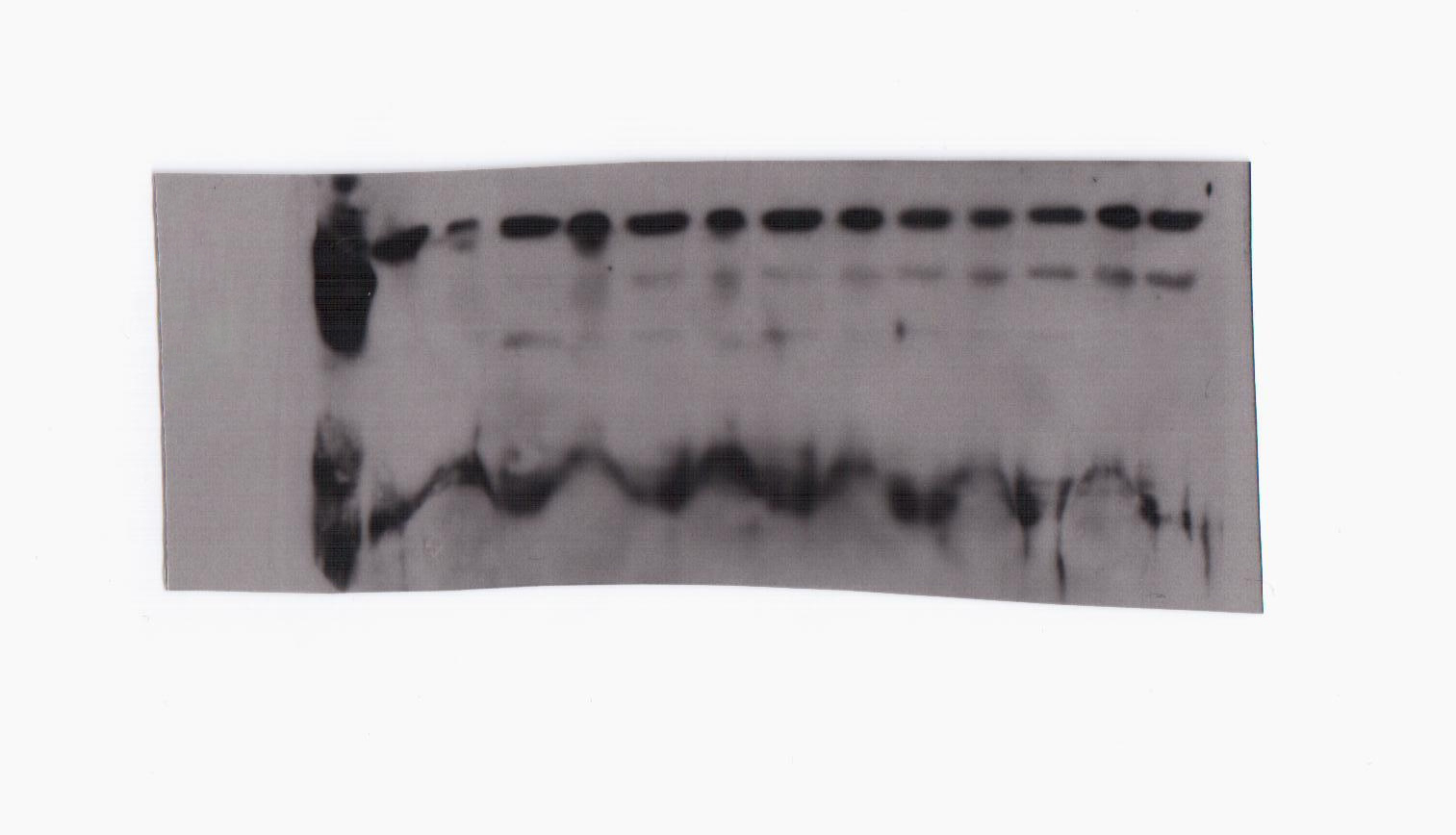

Supplement: Supplementary file 1 [file Image1.jpeg]
